# Risk Prediction of Colon Cancer Metastasis and Bioinformatics Analysis of Aspirin Treatment

**DOI:** 10.1155/ijog/8412254

**Published:** 2025-12-17

**Authors:** Jing Li, Xinyue Yu, Jingjing Shao, Hui Zhang, Jiawei Yu

**Affiliations:** ^1^ Research Center of Clinical Medicine, Affiliated Hospital of Nantong University, Nantong, Jiangsu, China, ahnmc.com; ^2^ School of Pharmacy, Nantong University, Nantong, Jiangsu, China, ntu.edu.cn; ^3^ Cancer Research Center Nantong, Affiliated Tumor Hospital of Nantong University & Nantong Tumor Hospital, Nantong, Jiangsu, China; ^4^ Clinical Laboratory, Affiliated Tumor Hospital of Nantong University & Nantong Tumor Hospital, Nantong, Jiangsu, China; ^5^ Gastrointestinal Surgery Department, Affiliated Hospital of Nantong University, Nantong, Jiangsu, China, ahnmc.com

**Keywords:** aspirin chemoprophylaxis, colon cancer, metastasis risk, prognosis

## Abstract

**Background:**

Metastasis is a major adverse prognostic factors of colon cancer. Aspirin chemoprophylaxis may improve outcomes for metastatic colon cancer patients. This study aimed to determine the impact of metastasis‐related molecular subtypes on prognosis and aspirin chemoprophylaxis benefit.

**Methods:**

We obtained differentially expressed metastasis‐related genes in cancer and normal tissues. A weighted gene co‐expression network (WGCNA) was constructed by differentially expressed genes. Lasso‐Cox regression identified key prognostic genes within relevant modules, establishing a risk score model. Transcription factors regulating module genes were explored. Aspirin‐interacting genes were identified using the Comparative Toxicogenomics Database (CTD) and validated via cellular experiments.

**Results:**

WGCNA analysis of 2062 metastasis‐related genes revealed significant correlations between blue/yellow modules and colon cancer. A risk score model based on blue module genes predicted overall survival and 1‐, 3‐, and 5‐year survival rates. Transcription factor analysis implicated the E2F family in blue module regulation and NF*κ*B1/STAT3 in yellow module regulation. CTD analysis showed persistent upregulation of NOX4, CXCL8, CXCL5, GDF15, and MMP13 post‐aspirin treatment. Cellular experiments confirmed aspirin downregulated metastasis‐related genes (E2F1, CCNE1, VEGFA, MMP3) in colon cancer.

**Conclusion:**

We developed a validated metastasis gene predictive model. Colon cancer patients with upregulated NOX4, CXCL8, CXCL5, GDF15, or MMP13 may not benefit from aspirin chemoprophylaxis. Conversely, patients showing aspirin‐induced downregulation of E2F1, CCNE1, VEGFA, and MMP3 may derive chemoprophylactic benefit.

## 1. Introduction

Global 2022 cancer statistics ranked colorectal cancer (CRC) third in incidence and second in mortality worldwide [1]. Dietary changes have significantly increased CRC incidence in China, with 376,300 new cases and 191,000 deaths reported in 2015 [2]. Despite improved survival rates with surgery and chemoradiotherapy, recurrence and metastasis remain major challenges, accounting for > 90% of cancer‐related deaths [3]. Tumor metastasis involves multi‐step, multi‐pathway, and multi‐gene alterations, therefore necessitating novel metastasis‐associated molecules and mechanisms to identify prognostic markers or therapeutic targets. In this study, we used the COAD dataset from the TCGA public database to analyze the differential expression of metastasis‐related genes between colon cancer tissues and adjacent tissues. Then, a weighted gene co‐expression network analysis (WGCNA) was performed on the differentially expressed genes (DEGs) to explore gene modules highly associated with colon cancer. Subsequently, Gene Ontology (GO) and Kyoto Encyclopedia of Genes and Genomes (KEGG) pathways were analyzed for genes of highly correlated modules. Protein–protein interaction (PPI) network and hub gene extraction analysis were used to further narrow the range of differential genes. Finally, we identified the major transcription factors that regulate key module genes associated with colon cancer. Together, our results provide a profound understanding of tumorigenesis, metastasis, and progression of colon cancer and identify central genes and upstream transcriptional regulators responsible for colon cancer prognosis.

In addition, although the treatment regimen of surgery combined with radiotherapy and chemotherapy has significantly improved the treatment of cancer patients, finding ways to further reduce the metastasis rate and mortality rate of colorectal cancer is still the focus of oncology research [4]. Chemoprophylaxis has become one of the most promising methods for cancer prevention and treatment, including the use of drugs, food, or nutritional ingredients to prevent the occurrence or reversal of precancerous lesions and prevent the development of existing precancerous lesions into invasive cancer [5]. Chemoprophylaxis of colorectal cancer is mainly associated with aspirin and metformin [4]. The data suggested that low‐dose aspirin could reduce the risk of colorectal cancer and could reduce the risk of death from the disease by limiting the spread of cancer cells [6]. But there are also potential dangers to taking aspirin. Low daily doses of aspirin irreversibly inhibit platelet activation, which may increase a patient’s risk of internal bleeding and allergic reactions [7]. Based on the occurrence of these side effects, our study aimed to understand which patients with colorectal cancer could benefit from aspirin treatment and which patients should stop aspirin trials immediately. In addition, a toxicogenomics approach is used to identify changes in the expression of genes after aspirin treatment and the key pathways involved.

## 2. Materials and Methods

### 2.1. Identification of Differentially Expressed Genes

First, we used “metastasis” as the keyword and searched for metastasis‐related genes on the GeneCards website on May 2022. We obtained a total of 10,061 genes. By conducting WENN analysis on these genes and the TCGA_COAD dataset, we ultimately obtained 8750 COAD‐related metastatic genes. Then, DEGs were identified using the SangerBox tools, a free online platform for data analysis (http://vip.sangerbox.com/). We evaluated the significance of each gene in the TCGA‐COAD dataset between tumor tissues and paracancerous tissues by using the R software package “t. Test” function, and calculated the significance FDR of each gene by using the P. adjust function, and finally obtained the difference information of each gene. During the screening process, the FDR value < 0.01 and |logFC| > 1.5 or < 1.5 were the cutoff standard.

### 2.2. Weighted Correlation Network Analysis (WGCNA)

WGCNA network construction was performed using the SangerBox tools. First, we calculated the MAD (median absolute deviation) of each gene by using the differential gene expression profile obtained from the TCGA‐COAD dataset. The first 50% of the smallest genes of MAD were removed, and the outliers and samples were removed by the good Samples Genes method of R software package “WGCNA,” and the scale‐free co‐expression network was further constructed by WGCNA. Specifically, at first, the Pearson’s correlation matrices and average linkage method were both performed for all pin‐wise genes. Then, a weighted adjacency matrix was constructed using a power function A_mn = | C_mn | ^ *β* (C_mn = Pearson ‘s correlation between Gene_m and Gene_n; A_mn = adjacency between Gene M and Gene N). *β* was a soft‐thresholding parameter that could protect strong correlations between genes and penalize weak correlations. The SangerBox tools use the WGCNA package of R language to automatically filter the best *β* value. Generally, when *β* is greater than 0.8 or the minimum *β* at the plateau stage is selected for network construction. After choosing the power of 6, the adjacency was transformed into a topological overlap matrix (TOM), which could measure the network connectivity of a gene defined as the sum of its adjacency with all other genes for gene network, and the corresponding dissimilarity (1‐TOM) was calculated. To classify genes with similar expression profiles into gene modules, average linkage hierarchical clustering was conducted according to the TOM‐based dissimilarity measure with a minimum size (gene group) of 100 for the gene dendrogram. Set sensitivity to 3. To further analyze the module, we calculated the dissimilarity of module eigen genes, chose a cut line for module dendrogram, and merged some modules. In addition, modules with a distance less than 0.25 were combined to obtain five co‐expression modules. It should be noted that grey module is considered as a gene set that cannot be assigned to any module.

### 2.3. Gene Enrichment Analysis

GO and KEGG pathway enrichment analyses of modulation‐related genes were analyzed and mapped in colon cancer using the SangerBox tools. KEGG pathway enrichment analyses: For enrichment of gene set function analysis, we use KEGG rest API (https://www.kegg.jp/kegg/rest/keggapi.html) to obtain the latest KEGG pathway gene annotation as the background for mapping genes to the collection, and R software package “clusterProfiler” (Version 3.14.3) was used for enrichment analysis to obtain the results of gene set enrichment. GO enrichment analysis: For gene set functional enrichment analysis, we used the GO annotation of genes in R software package “org.hs.eg.db” (Version 3.1.0) as the background, mapped genes into the background set, and used R software package “clusterProfiler” (Version 3.14.3) for enrichment analysis. Enrichment analysis was performed using the R package "clusterProfiler" (Version 3.14.3). The minimum and maximum gene set sizes were 5 and 5000, respectively, with a significance threshold of *p* < 0.05 and FDR < 0.1.

### 2.4. PPI Network Construction and Cytoscape Software Analysis

The PPI network is a search tool used to retrieve interacting genes (STRING; http://string-db.org) online database [8]. In this study, PPI networks of module‐related genes were constructed using the STRING database, and interactions with composite scores greater than 0.7 were considered statistically significant. Cytoscape (version 3.8.2) is an open‐source bioinformatics software platform for visualizing networks of gene interactions. CytoHubba plugin was used to assign value to each gene through topological network algorithm, and the key gene (hub gene) and subnetwork were sorted and mined. To obtain more comprehensive information on the biology of module‐related genes, Cytoscape v3.8.2 and its plugins (CytoHubba, ClueGO v2.5.9, CluePedia v1.5.9, iRegulon) were used to visualize the biology of genes in a functionally grouped network [9, 10]. Cytoscape plugin iRegulon is used to predict transcription factors that regulate major genes in key modules [11].

### 2.5. Metascape

Metascape (http://metascape.org) was used to identify pathways and enrich genes associated with modules [12], including only those with a minimum overlap of 3,*p* value < 0.05, and minimum concentration of 3. The identified enriched ontology clusters were then transformed into a network layout.

### 2.6. Genetic Risk Score Model Construction

Univariate analysis and Lasso‐Cox analysis of blue module‐related genes were analyzed in colon cancer using the SangerBox tools. Univariate analysis: We used the R software package “Survival” to integrate survival time, survival status, and gene expression data and evaluated the prognostic significance of each gene by Cox method. Lasso‐Cox: We used the R software package “GlMNET” to integrate survival time, survival status, and gene expression data and performed regression analysis using Lasso‐Cox methods. In addition, we also set up 10‐fold cross‐validation to obtain the optimal model. The optimal *λ* value (0.00926506658015332) yielded 14 prognostic genes. The 14 genes were then used to construct a risk‐score equation. For Kaplan–Meier curves, *p* values and hazard ratio (HR) with 95% confidence interval (CI) were generated by log‐rank tests and univariate cox proportional hazards regression. A nomogram was established using Cox method to evaluate the prognostic significance of these features in 432 samples by integrating the data of survival time, survival status, and four characteristics (risk score, age, M, and stage).

### 2.7. Aspirin Interacting Genes

In this study, aspirin interacting genes were derived from the Comparative Toxicogenomics Database (CTD; http://CTD.mdibl.org), a publicly available and scientifically useful resource that contains comprehensive data to better understand the relationship between chemicals, genes, and disease [13]. Aspirin‐interacting genes were sourced from the CTD (June 2022 download).

### 2.8. Cell Culture and Salicylate Treatment

The CRC cell line LoVo cells were cultured in F12k medium with 10% fetal bovine serum (Gibco, Thermo Fisher Scientific) at 5% CO_2_ and 37°C. Sodium salicylate (Sigma‐Aldrich) was dissolved in ddH_2_O into a 600 mM stock solution and applied at a working concentration of 5 mM.

### 2.9. Wound Healing Assay

LoVo cells were seeded into six‐well plates at the density of 1 × 10^6^ cells/mL. After 24 h of culture, the single‐layer cells in each well were scratched with a yellow tip to form a wound. After washing the cells with phosphate‐buffered saline for three times, the cells were treated with 5 mM sodium salicylate for 24 h and photographed under the microscope either immediately (*t* = 0 h) or 24 h later (*t* = 24 h). Cell migration was assessed by measuring wound gap sizes in at least six areas. Each experiment was repeated three times.

### 2.10. Transwell Assay

To assess the migration capacity of LoVo cells, analysis was performed using a polycarbonate membrane transwell chamber containing a filter with a diameter of 6.5 mm and a pore size of 8 *μ*m. Then, LoVo cells were treated with 5 mM sodium salicylate for 48 h. LoVo cells in the polycarbonate filter were fixed with 4% paraformaldehyde and stained with 0.4% crystal violet. LoVo cells opposite the filter are photographed under an optical microscope (200× magnification).

### 2.11. RNA Isolation and Real‐Time Quantitative Polymerase Chain Reaction (RT‐qPCR)

Total RNA was extracted from LoVo cells by TRIzol reagent (Takara, Japan). One microgram of RNA was reverse‐transcribed into cDNA using a reverse transcription kit (Thermo Fisher Scientific, United States). RT‐qPCR was performed using SYBR Premix Ex TaqII (Takara, Japan) and ABI StepOnePlus (Applied Biosystems Inc., United States) systems. Relative mRNA expression was calculated by 2^−*ΔΔ*Ct^ cycle. The expression of each target mRNA was normalized to GAPDH. Primer sequences (Sangon Biotech): E2F1, 5 ^′^‐GGATTTCACACCTTTTCCTGGAT‐3 ^′^ (forward) and 5 ^′^‐CCTGGAAACTGACCATCAGTACCT‐3 ^′^ (reverse); CCNE1, 5 ^′^‐TACACCAGCCACCTCCAGACAC‐3 ^′^ (forward) and 5 ^′^‐CCTCCACAGCTTCAAGCTTTTG‐3 ^′^ (reverse); VEGFA, 5 ^′^‐AGGGCAGAATCATCACGAAGT‐3 ^′^ (forward) and 5 ^′^‐AGGGTCTCGATTGGATGGCA‐3 ^′^ (reverse); MMP3, 5 ^′^‐AGTCTTCCAATCCTACTGTTGCT‐3 ^′^ (forward) and 5 ^′^‐TCCCCGTCACCTCCAATCC‐3 ^′^ (reverse); GAPDH, 5 ^′^‐GCACCGTCAAGGCTGAGAAC‐3 ^′^ (forward) and 5 ^′^‐TGGTGAAGACGCCAGTGGA‐3 ^′^ (reverse).

### 2.12. Statistical Analysis

For univariate and multivariate analyses, SPSS 25.0 software (SPSS Inc, Chicago, United States) was used. A *p* < 0.05 was recognized as statistical significance.

## 3. Results

### 3.1. Differential Gene Identification and Pathway Enrichment

**Figure 1:**
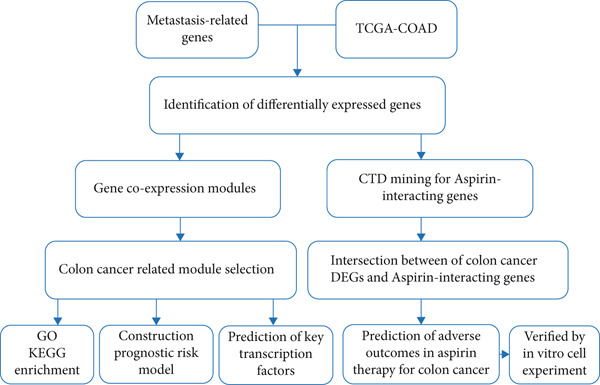
Flowchart of analysis.

We used bioinformatics analysis to elucidate the prognostic value, potential regulatory pathways, and molecular mechanisms of metastatic genes in colon cancer, with the overall analysis flowchart depicted in Figure 1. Specifically, we obtained 8750 metastatic genes from GeneCards (Figure 2a). First, we evaluated metastasis gene expression profiles in the TCGA‐COAD samples, which included 456 colon cancer samples and 41 normal samples. We identified 2062 DEGs (1072 upregulated, 990 downregulated) between 456 colon cancer and 41 normal samples (Figure 2b). Subsequently, we conducted the following analysis based on this part of the data.

Figure 2Identification of differential metastasis genes from TCGA‐COAD database. (a) Venn diagram of metastasis gene mRNA in GeneCards. (b) A volcanic map of differential genes. Red dots represent up‐regulated genes and green dots represent down‐regulated genes. (c) Heat maps of different genes.

**Figure:**
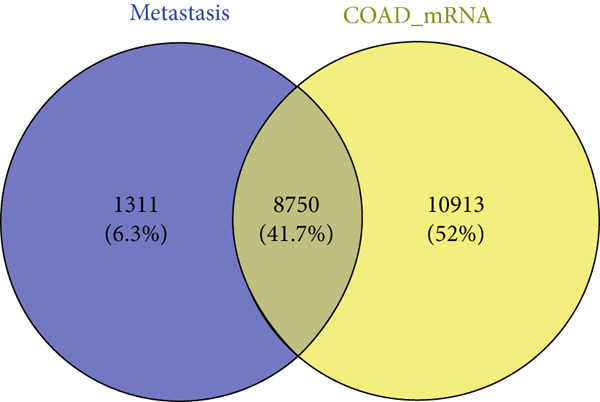
(a)

**Figure:**
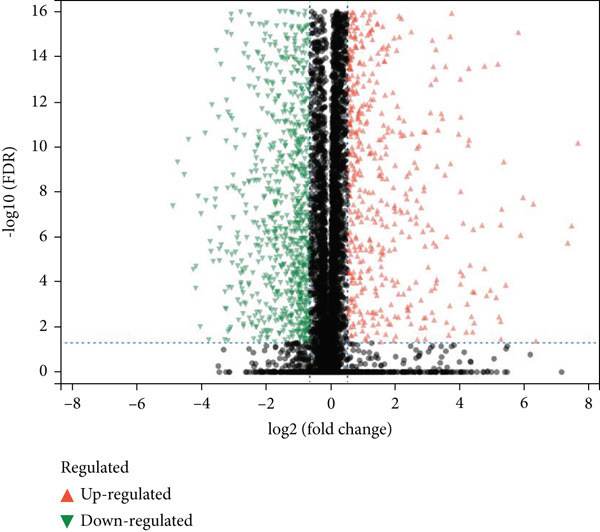
(b)

**Figure:**
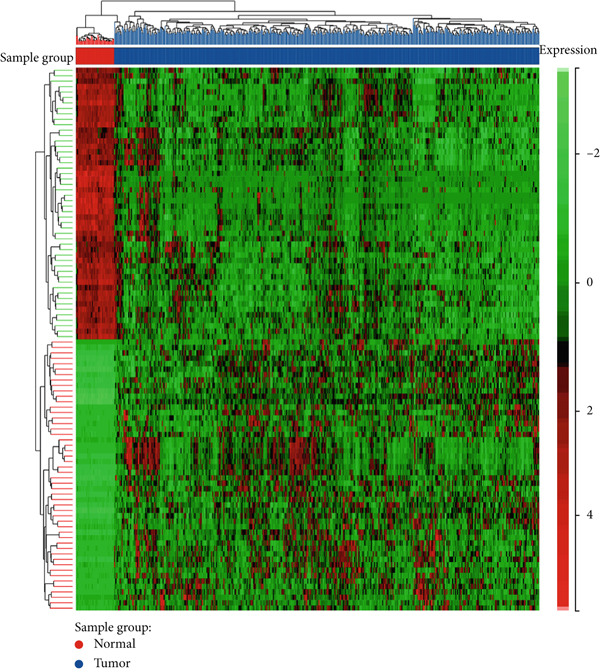
(c)

### 3.2. Construction of WGCNA Network

We constructed WGCNA co‐expression network using 2062 genes to identify modules highly associated with colon cancer. When the power value (*β*) was 6 and the scale‐independent value was 0.88, the average connectivity of these genes was high, and the connectivity between genes was in accordance with the scale‐free network distribution (Figure 3a,b). Therefore, we chose *β* = 6 to generate a hierarchical clustering tree, with different colors representing different modules. All the genes with similar expression patterns were grouped into modules through mean linkage clustering (Figure 3c). Five different gene modules (blue, brown, turquoise, yellow, and gray) were identified and displayed in different colors, with the gray module considered to be a set of genes that could not be assigned to any module. The neighbor connection network and phylogenetic clustering diagram of characteristic genes are shown in Figure 4a,b. Blue and yellow modules were positively associated with colon cancer (*p* < 0.001), while brown and turquoise modules were significantly negatively correlated with colon cancer (Figure 4c).

Figure 3Identification of key gene by WGCNA. (a) Scale independence and (b) mean connectivity analysis of various soft‐threshold powers. (c) Gene clustering dendrogram based on difference measure (1 − TOM), with differences based on topological overlap.

**Figure:**
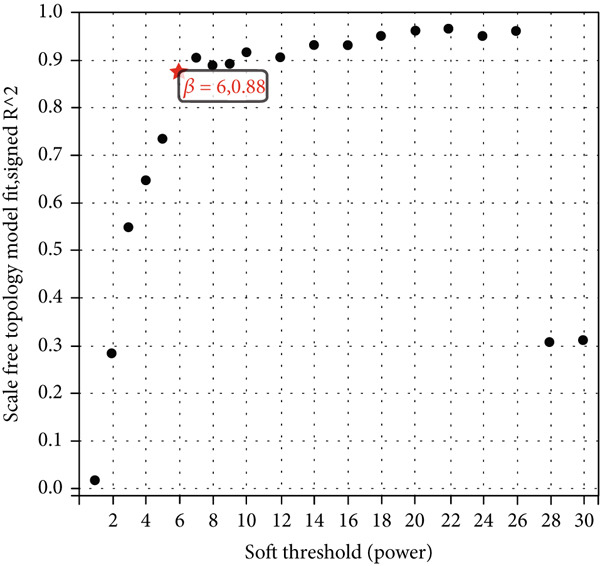
(a)

**Figure:**
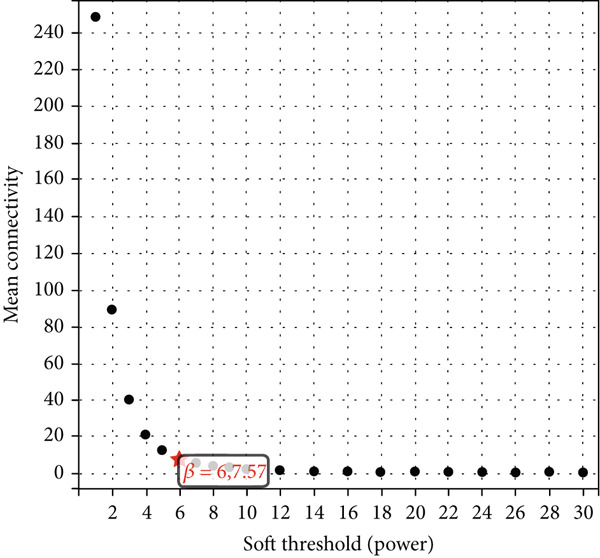
(b)

**Figure:**
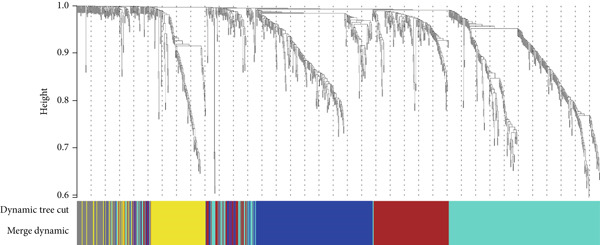
(c)

Figure 4Module–trait correlations analyses. (a) The heat map plot of the interactions among co‐expression modules. (b) Module–trait relationships. (c) Scatter diagram of membership degree between module and colon cancer and normal tissues.

**Figure:**
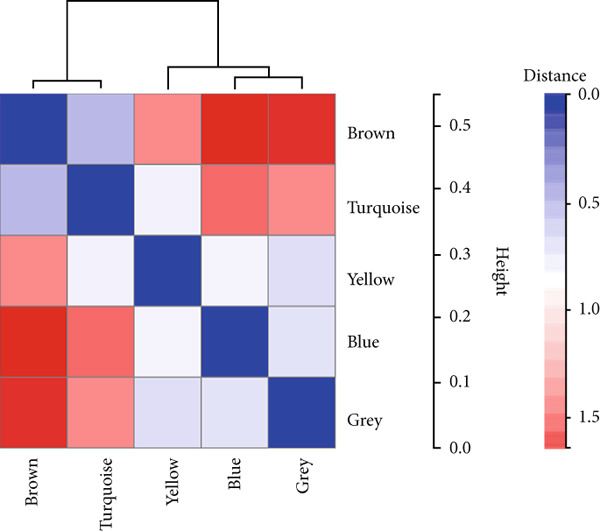
(a)

**Figure:**
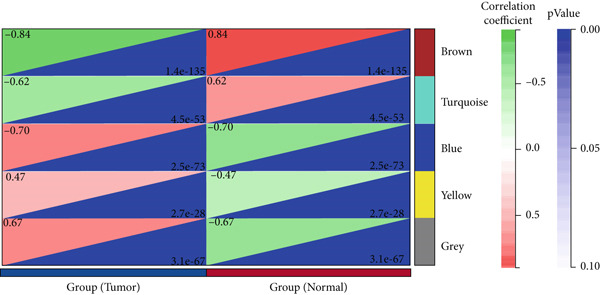
(b)

**Figure:**
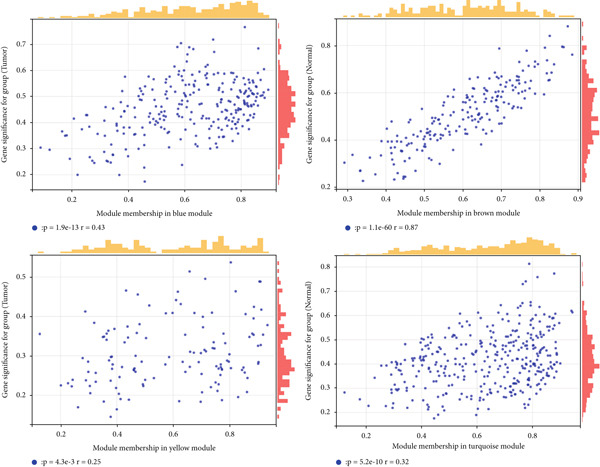
(c)

### 3.3. Cell Cycle‐Associated Metastasis Genes Are Associated With Colon Cancer Progression

Firstly, we performed GO analysis and KEGG pathway enrichment analysis on the genes in the blue module to explore the key biological processes and signaling pathways involved in the progression of colon cancer with tumor metastasis genes. GO analysis and KEGG pathway enrichment results showed that genes in the blue module were mainly highly correlated with cell cycle (Figures 5a, 5b, 5c, and 5d). In addition, GO analysis was found to be associated with chromatin remodeling (Figure 5a). Metascape GO enrichment results also confirmed the above results (Figure 5e).

Figure 5Significantly enriched KEGG pathways and GO annotations of blue module. The KEGG pathways and GO annotations of blue module genes were analyzed by GSEA. The top 10 significant networks were chosen. (a) KEGG pathway analysis; (b) GO‐BP; (c) GO‐CC; (d) GO‐MF. (e) Network of enriched terms by Metascape colored by *p* values.

**Figure:**
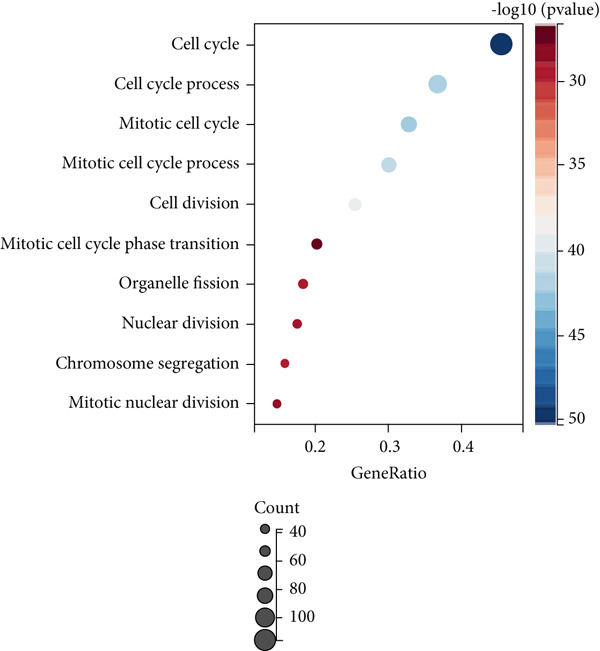
(a)

**Figure:**
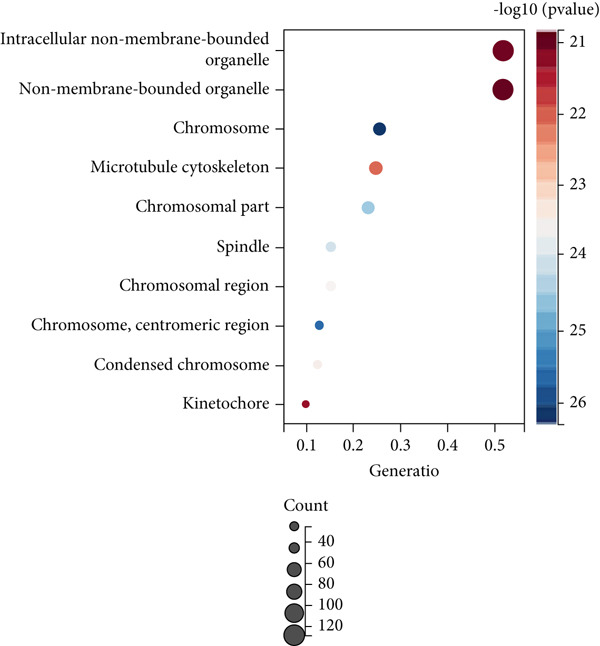
(b)

**Figure:**
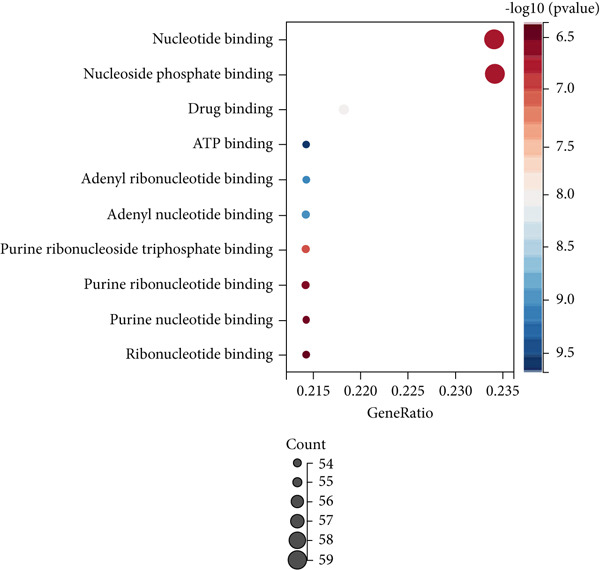
(c)

**Figure:**
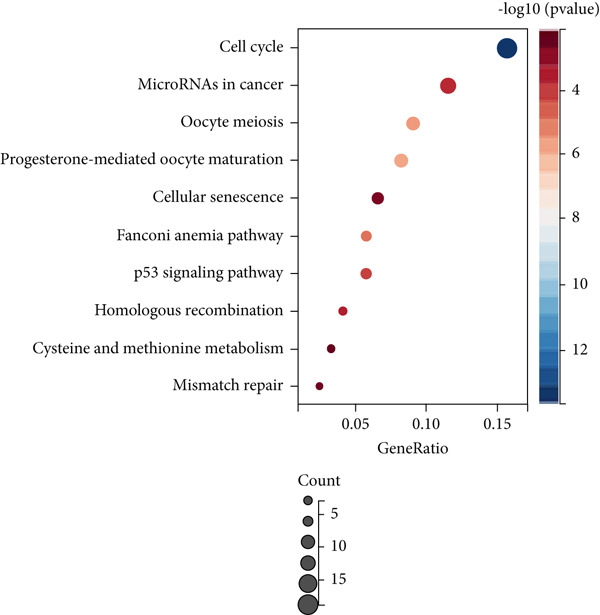
(d)

**Figure:**
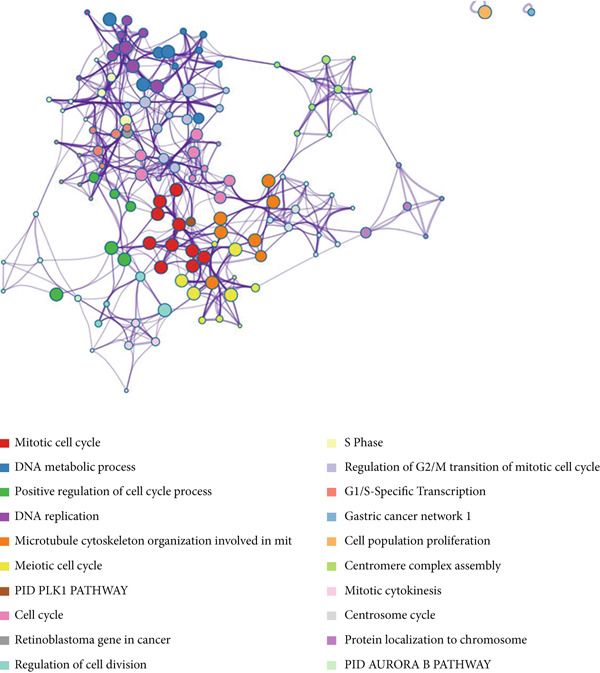
(e)

Two hundred sixty‐three genes in the blue module were selected to map the PPI network. Hub genes were extracted by Cytoscape software, and the top 50 genes most related to colon cancer were finally determined (Figure 6a,b). The degree value of each node in PPI interactive network was calculated and sorted. Nodes with degree value greater than 30 are located on the right side of Figure 6b, mainly including 11 genes (CDK1, TTK, CENPF, TPX2, NCAPG, DLGAP5, AURKA, KIF20A, BUB1, BUB1B, ASPM).

Figure 6Gene interaction network diagram in blue module. (a) PPI network analysis of blue module. (b) Network of top 50 hub genes in blue module.

**Figure:**
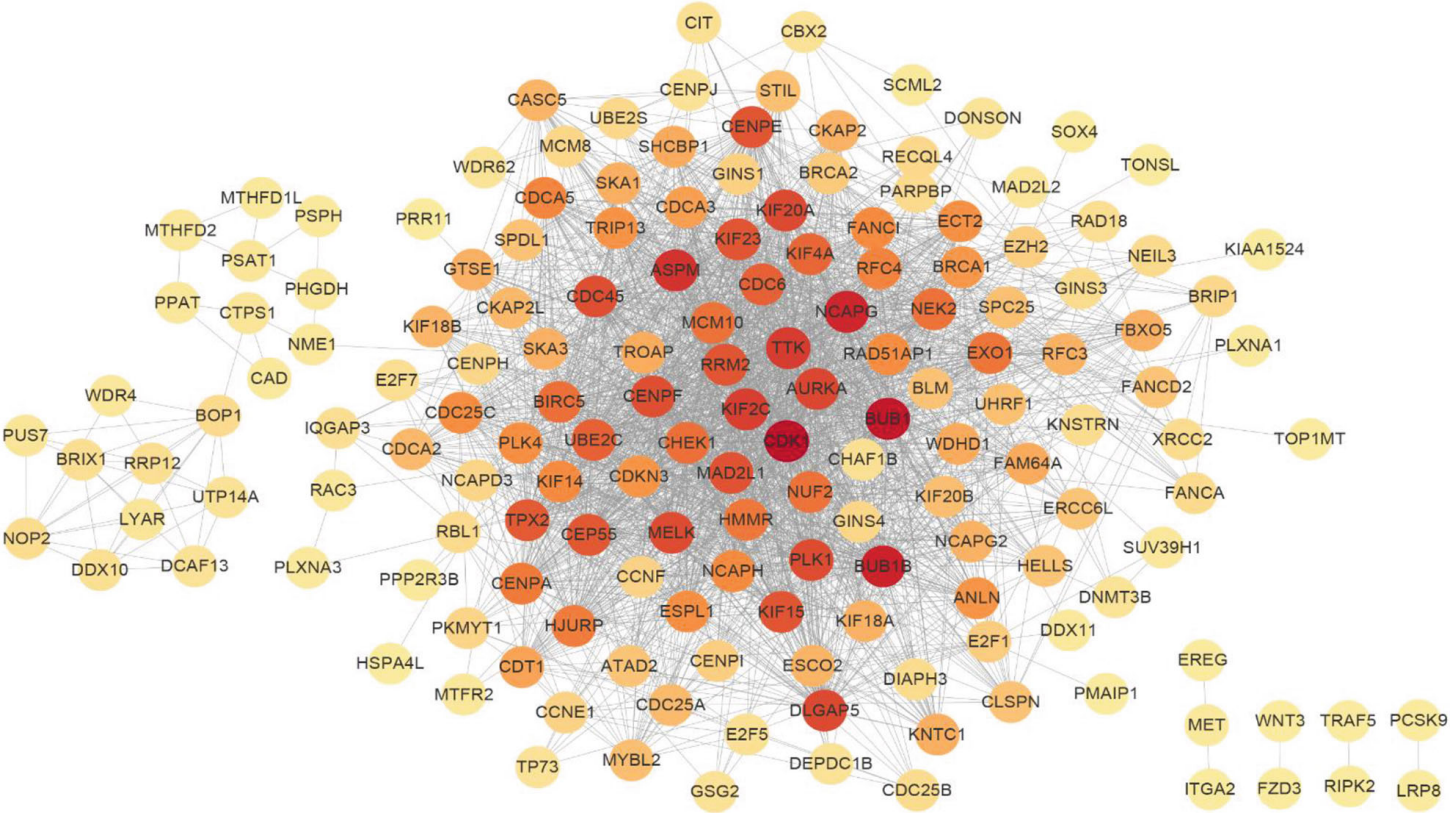
(a)

**Figure:**
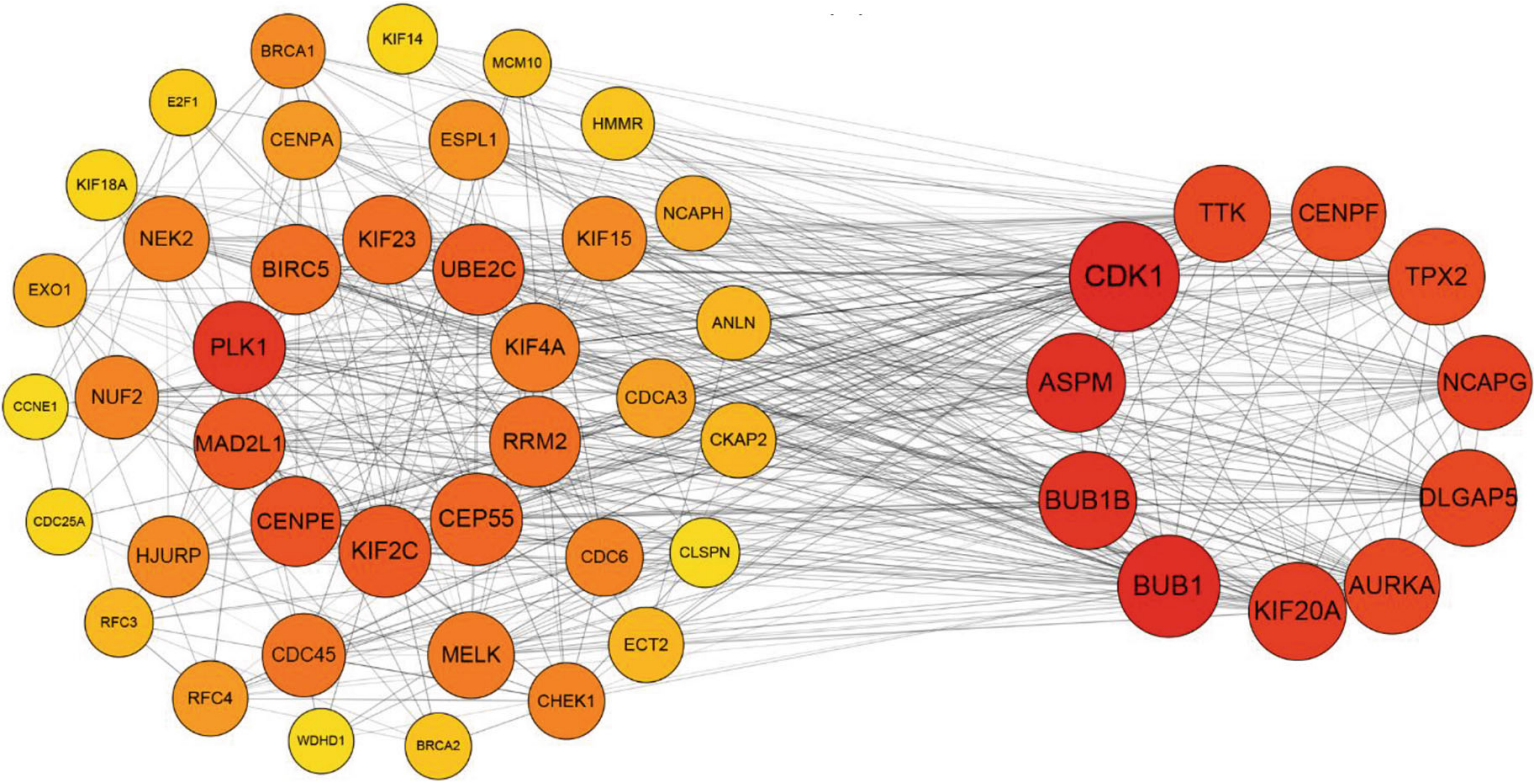
(b)

Subsequently, the Metascape database was used for GO‐BP analysis of top 50 hub genes (Figure 7a). We found that the results are enriched by many cell cycle‐related biological events. This is consistent with the previous results of gene enrichment in blue module. Next, the biological functions of the common genes and their co‐expressed genes were analyzed using ClueGO and CluePedia, and they showed rich functions in mitotic cell cycle phase transition, nuclear division, regulation of cell cycle process, and chromosome organization (Figure 7b). Pathway enrichment analysis showed that blue module genes were mainly divided into nine clusters, including cell cycle, microRNA in cancer, cellular senescence, p53 signaling pathway, mismatch repair, Fanconi anemia pathway, homologous recombination, progesterone‐mediated oocyte maturation, and oocyte meiosis (Figure 7c).

Figure 7Functional enrichment analysis of hub gene of blue module. (a) Bar graph of enriched terms across input gene lists, colored by *p* values. (b) GO network of hub genes. (c) Pathways analysis of hub genes.

**Figure:**
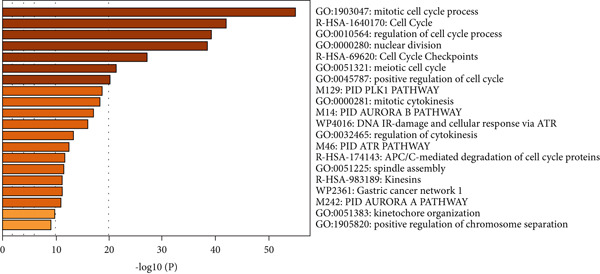
(a)

**Figure:**
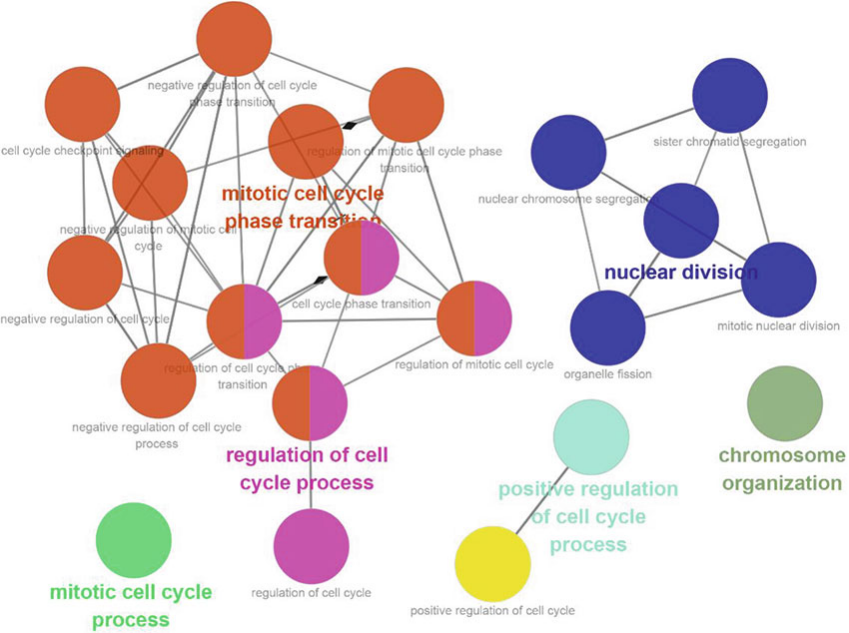
(b)

**Figure:**
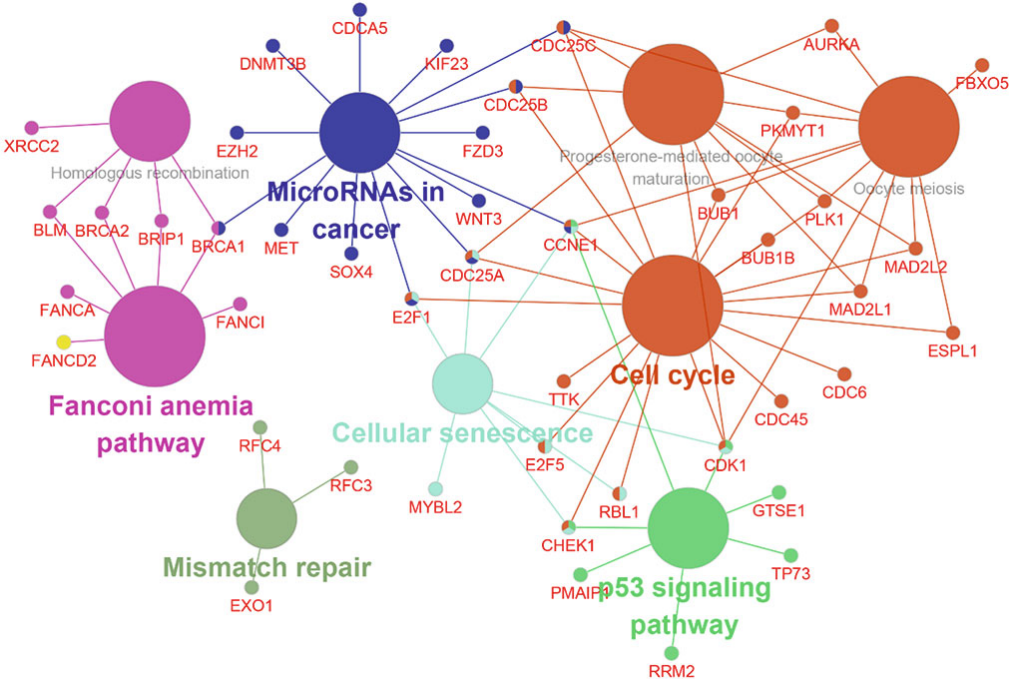
(c)

Next, we analyzed the key regulatory transcription factors of the blue module hub gene using Metascape database (Figure 8a). The results showed that the blue module hub gene was mainly regulated by E2F family transcription factors. In addition, we also used iRegulon plugin in Cytoscape software to predict transcription factors of blue module hub gene and compared the obtained transcription factors with Metascape database analysis results. Finally, two main transcription factors were obtained as E2F1 and E2F4. By plotting the transcription factor regulatory network, we found that E2F1 and E2F4 can regulate most blue module hub genes, such as CDK1, BUB1, BUB1B, TTK, ASPM, NCAPG, and so on (Figure 8b).

Figure 8Transcription factor prediction of the hub gene in the blue module. (a) Summary of enrichment analysis in TRRUST. (b) The regulatory network of major transcription factors and target genes in the blue module.

**Figure:**
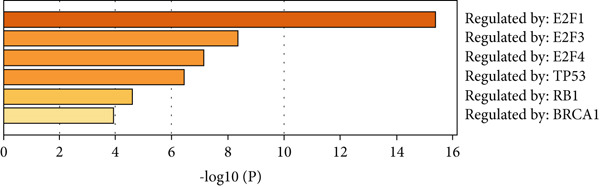
(a)

**Figure:**
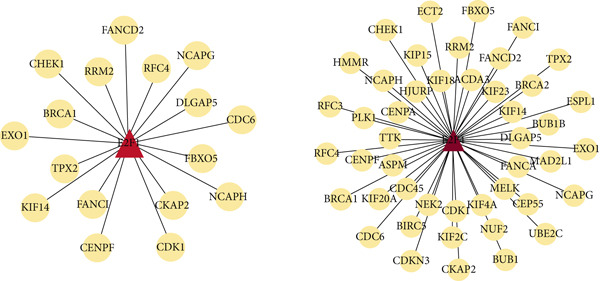
(b)

### 3.4. Risk Score Model Construction

**Figure 9:**
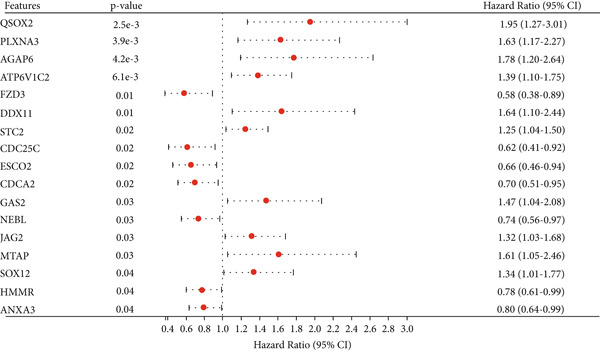
Univariate analysis of blue module gene.

We further investigated the prognostic value of differential metastasis genes in colon cancer in the TCGA‐COAD dataset. Firstly, we screened out genes significantly associated with the prognosis of colon cancer by univariate COX regression analysis. As shown in Figure 9, 17 genes were obtained with *p* values < 0.05. Then, according to the results of univariate Cox regression analysis, the excess genes were removed by Lasso regression analysis to obtain the genes with prognostic significance (Figure 10a,b). Three genes were removed, and the remaining 14 genes were further analyzed as QSOX2, PLXNA3, AGAP6, ATP6V1C2, FZD3, DDX11, STC2, CDC25C, CDCA2, GAS2, NEBL, MTAP, SOX12, and HMMR. Multivariate Cox regression analysis was performed to construct the final prognostic model of metastasis genes associated with colon cancer. Each patient’s risk score was calculated using a risk score model. Heat maps were generated to directly show the relationship between differential genes and risk scores (Figure 10c). Next, we used survival analysis to determine the predictive value of risk score for patients’ prognosis. A 14‐gene risk score stratified patients into high‐/low‐risk groups (cutoff = 1.343). High‐risk patients had poorer OS (HR = 3.617, *p* < 0.001; Figures 10d and 11b). The median survival time of high‐risk group was 1106 days based on Kaplan–Meier analysis.

Figure 10Lasso‐Cox regression analysis of 17 genes in the blue module. (a) The solution of Lasso. (b) Partial log‐likelihood profile of Lasso. (c) Heat maps of 14 prognostic gene expression profiles in high‐risk and low‐risk groups of TCGA‐COAD. (d) Kaplan–Meier analysis of high‐ and low‐risk groups from TCGA‐COAD.

**Figure:**
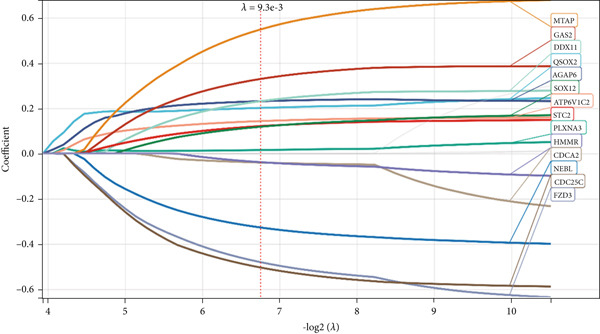
(a)

**Figure:**
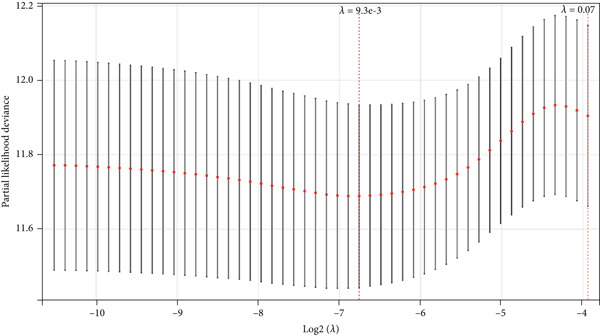
(b)

**Figure:**
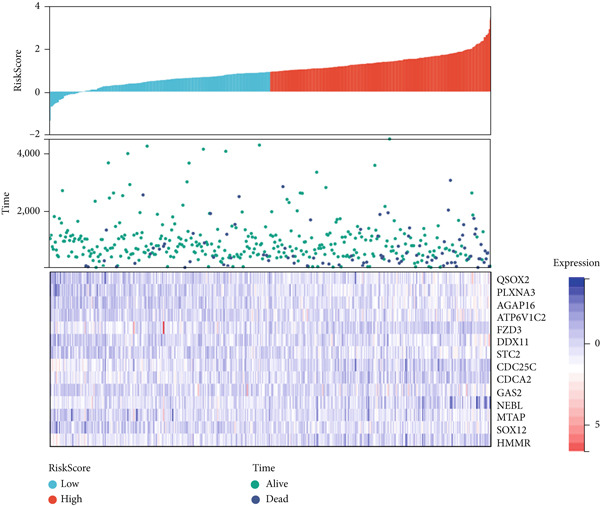
(c)

**Figure:**
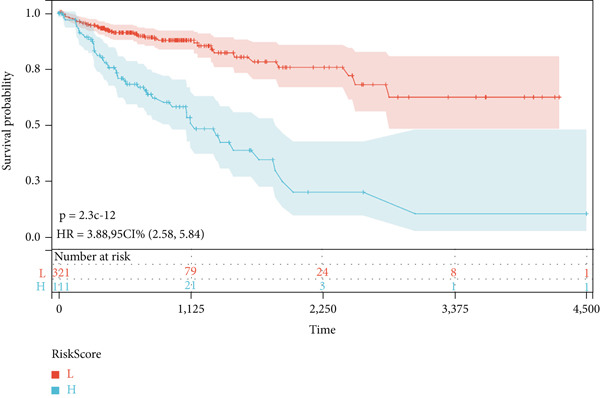
(d)

Figure 11Prognostic prediction of risk score. (a) Univariate Cox regression analysis of the colon cancer cohort. (b) Multivariate Cox regression analysis of the colon cancer cohort.

**Figure:**
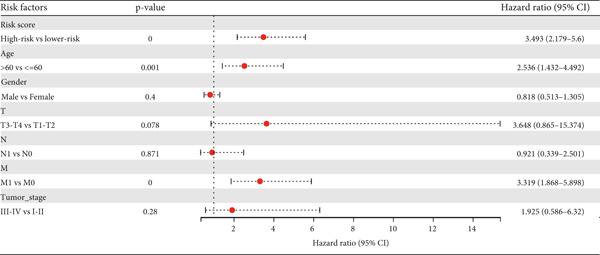
(a)

**Figure:**
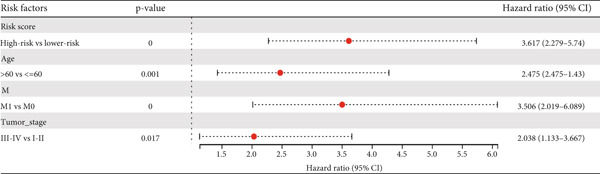
(b)

Next, we further evaluated the prognostic value of the constructed risk score in combination with clinical and pathological indicators such as age, sex, TNM stage, and tumor stage. Univariate COX regression analysis showed that risk score was significantly correlated with overall survival (HR = 3.493, 95*%*CI = 2.179–5.6, *p* value < 0.001) (Figure 11a), and multivariate COX regression analysis showed that risk score was an independent prognostic indicator of colon cancer as well as age, M stage, and tumor stage (HR = 3.617, 95*%*CI = 2.279–5.74, *p* value < 0.001) (Figure 11b). Nomogram and calibration maps were used to quantify the contribution of individual factors to clinical prognosis and validate the validity of the model (Figure 12a,b). The results show that the prediction model has good predictive ability. The area under the ROC curve of the model indicates its ability to predict the prognosis of colon cancer (Figure 12c). Moreover, by comparing with the ROC curve of tumor stage (the gold standard for colorectal cancer prognosis in clinical settings), the predictive model that includes risk scores has a more effective predictive ability for the survival rate of colon cancer patients, especially for 1‐year and 3‐year survival (Figure 12c,d). These results indicate that the prediction model has good prediction ability in colon cancer.

Figure 12Prediction of the prognosis probability in colon cancer. (a) Nomogram in the colon cancer cohort. (b) Colon cancer cohort calibration map. (c) ROC curve for the prediction model with risk score, age, M stage, and tumor stage in TCGA‐COAD. (d) ROC curve for tumor stage in TCGA‐COAD.

**Figure:**
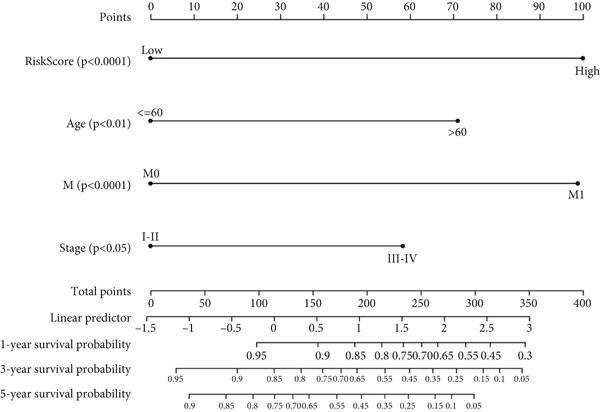
(a)

**Figure:**
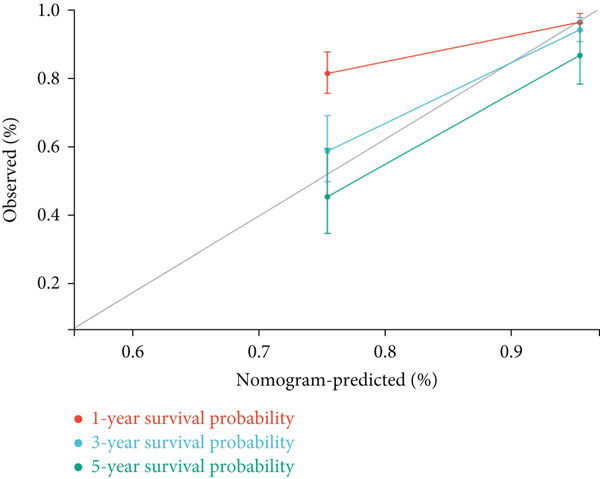
(b)

**Figure:**
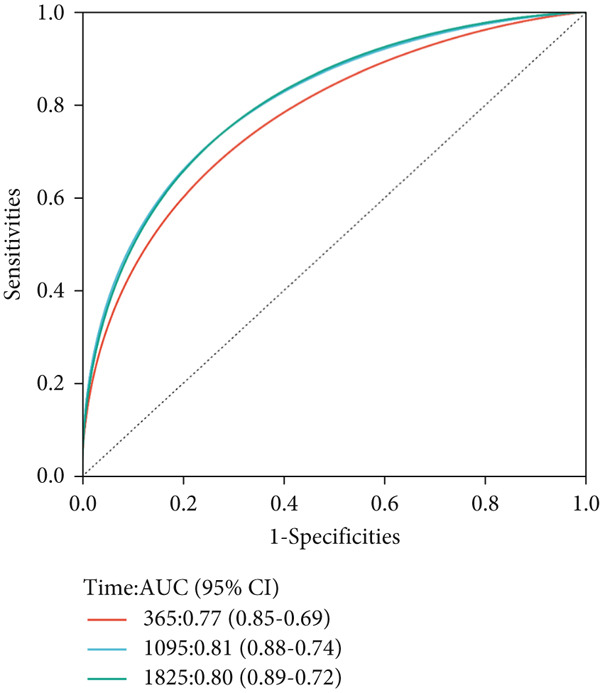
(c)

**Figure:**
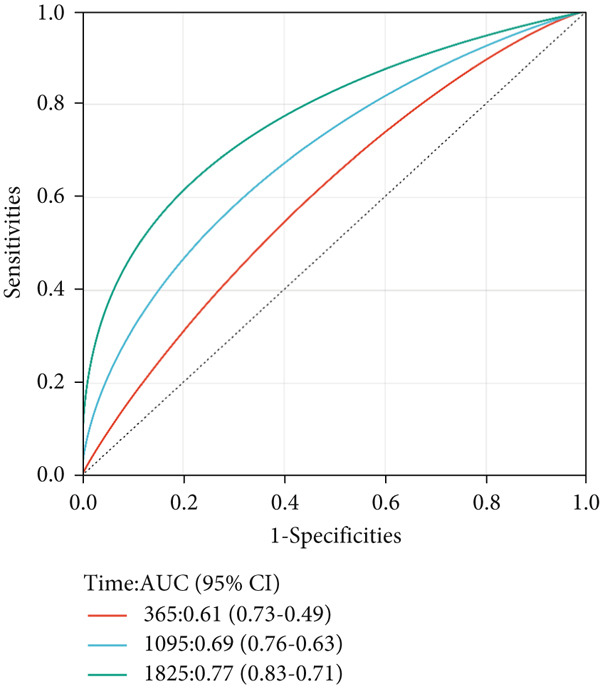
(d)

### 3.5. Co‐Expression Modules Associated With Metastasis of Colon Cancer

Analysis of genes in the yellow module revealed the module most closely associated with colon cancer metastasis (Figure 13). Genes in the module were enriched in pathways related to extracellular matrix organization, regulation of angiogenesis, and cell adhesion processes. The top 50 hub genes in this module were extracted, and upstream transcription factors were predicted using Metascape database. It was found that these key genes were mainly regulated by RELA, NF*κ*B1, JUN, STAT3, and other transcription factors (Figure 14a). Combined with the predicted results of iRegulon, Cytoscape software plugin, two common and reliable transcription factors, NF*κ*B1 and STAT3, were obtained. Figures 14b and 14c show the network diagram of yellow module genes regulated by these two transcription factors.

Figure 13Functional enrichment analysis of hub gene of yellow module. (a) Bar graph of enriched terms across input gene lists, colored by *p* values. (b) GO network of hub genes. (c) Pathways analysis of hub genes.

**Figure:**
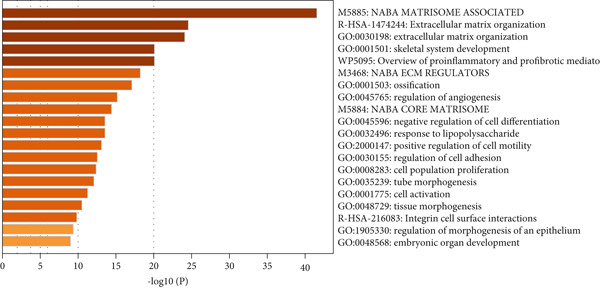
(a)

**Figure:**
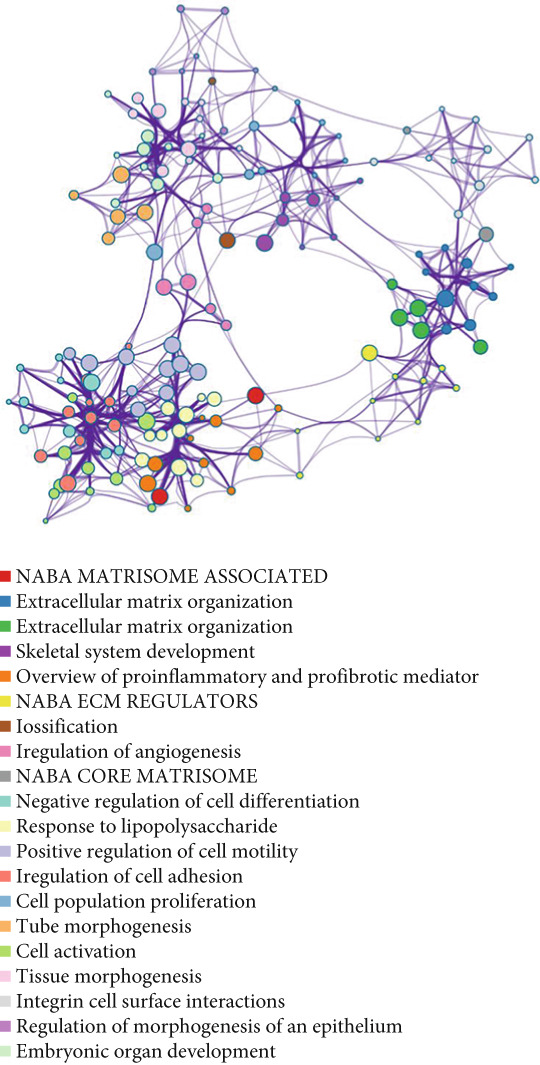
(b)

**Figure:**
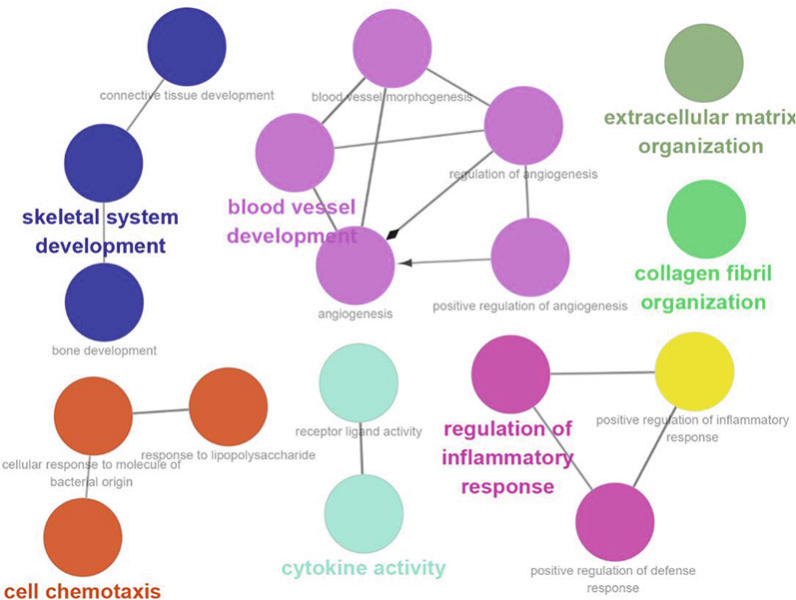
(c)

**Figure:**
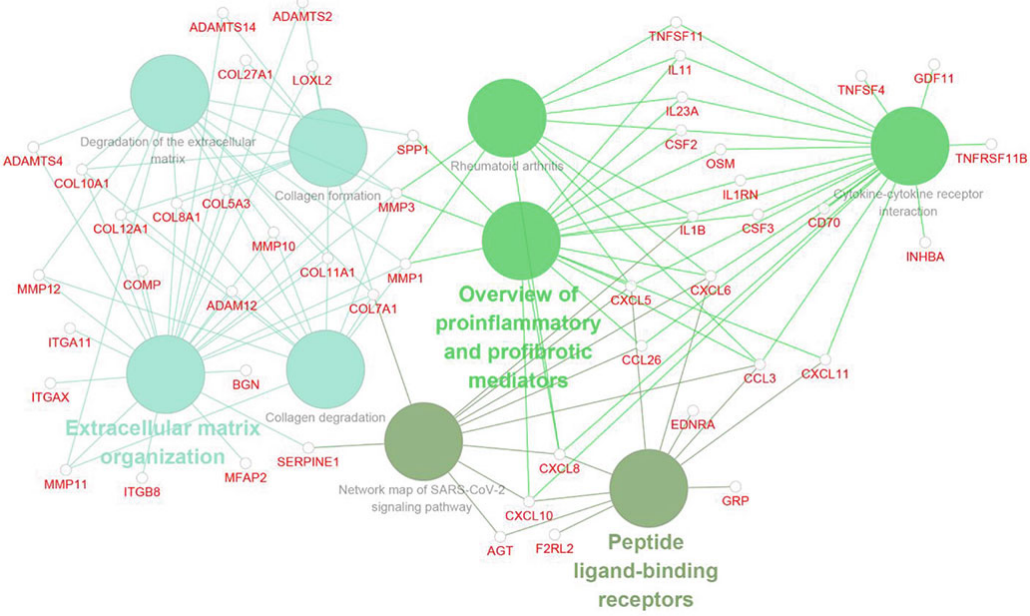
(d)

Figure 14Transcription factor prediction of the hub gene in the yellow module. (a) Summary of enrichment analysis in TRRUST. (b) The regulatory network of transcription factor NF*κ*B1 and its target genes. (c) The regulatory network of transcription factor STAT3 and its target genes.

**Figure:**
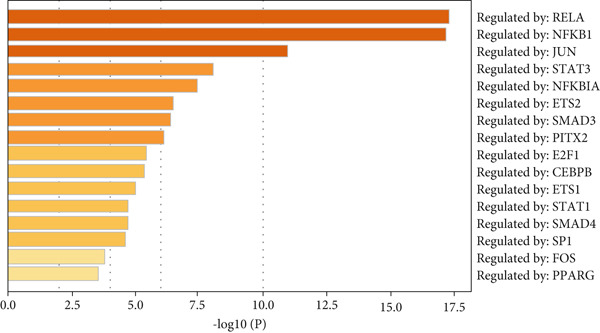
(a)

**Figure:**
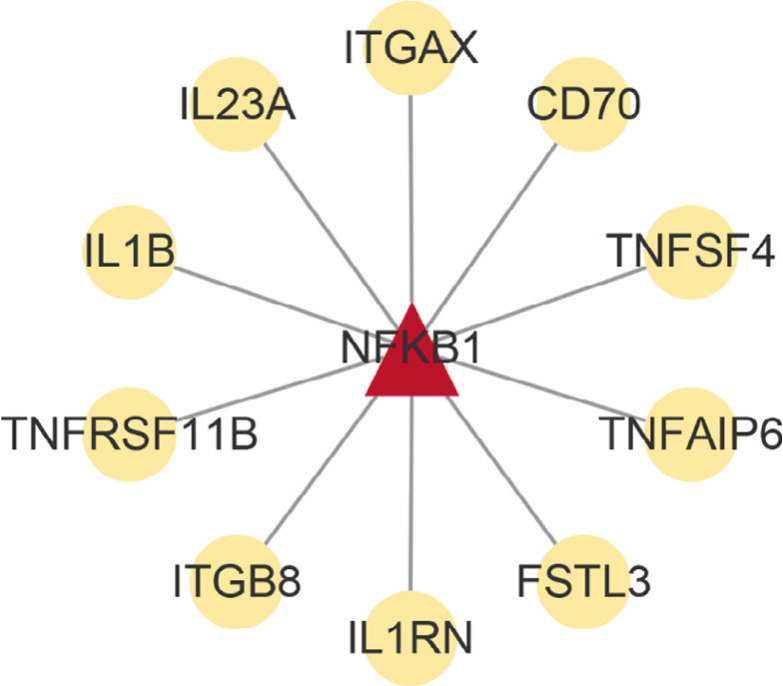
(b)

**Figure:**
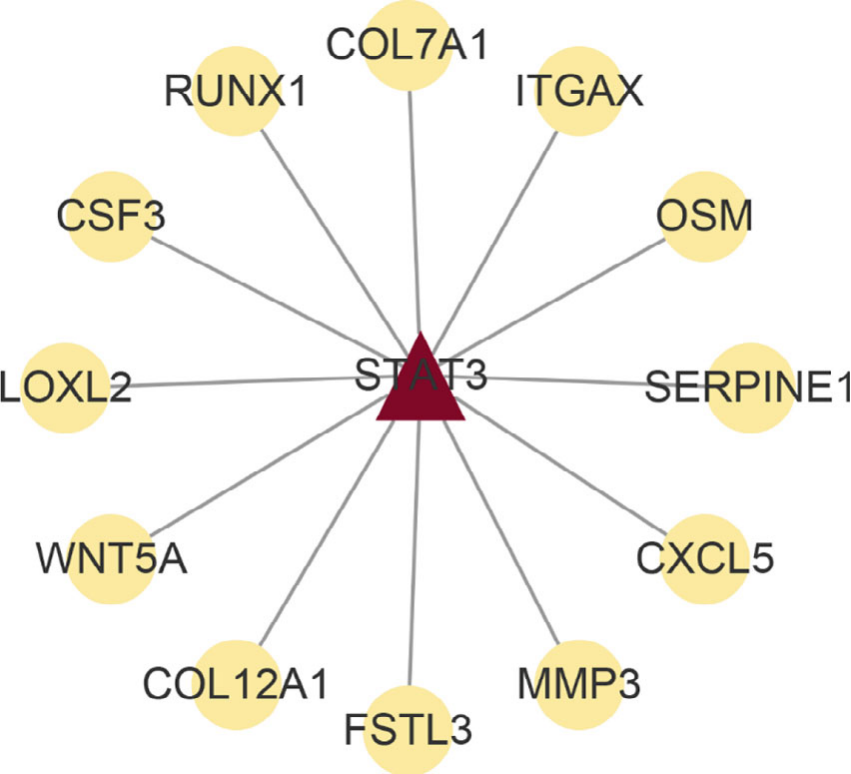
(c)

### 3.6. Aspirin Interacting Genes

Aspirin interacted with 720 genes (source: CTD), of which 126 genes were differentially expressed in colon cancer patients.

**Figure 15:**
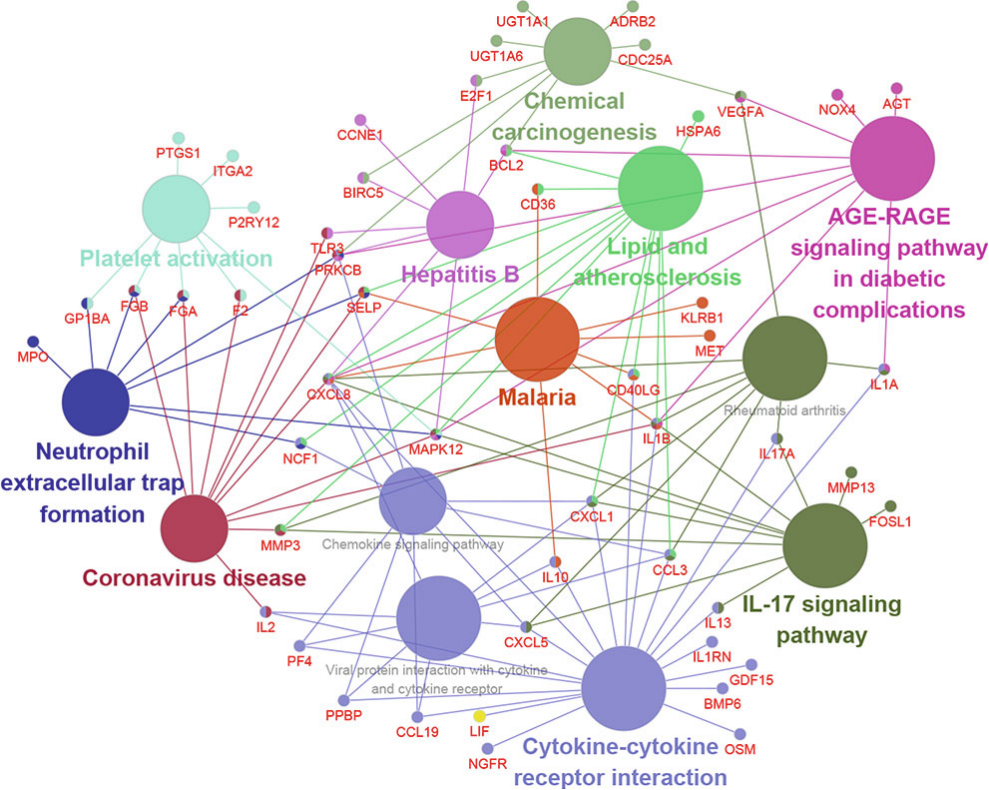
Pathway analysis of aspirin interaction dysregulation genes in colon cancer. Node size indicates the percentage of genes involved in the regulation of each molecular pathway. Colors indicate that genes are associated with specific molecular pathways.

The 126 colon cancer DEGs were analyzed by pathway enrichment analysis (ClueGo+CluePedia). The results showed that 38 out of 126 genes were enriched in platelet activation, chemical carcinogenesis, neutrophil extracellular trap formation, cytokine–cytokine receptor interaction, IL‐17 signaling pathway, AGE‐RAGE signaling pathway in diabetic complications, coronavirus disease, chemokine signaling pathway, and other signaling pathways (Figure 15). In addition, all 38 genes were associated with metastasis of colon cancer.

CTD chemical‐gene query were used to identify regulatory relationships between aspirin and 38 genes in the gene set associated with the prediction pathway (Table 1). The genes that were up‐regulated or down‐regulated by aspirin were then compared with their differential expression (compare the tumor tissue with the adjacent tissue) in colon cancer. This approach helps uncover the interactions between aspirin and important genes related to prognosis or metastasis. We found that aspirin further upregulated NOX4, CXCL8, CXCL5, LIF, GDF15, and MMP13, while stimulated inhibition of IL2, NCF1, and PTGS1. Interestingly, aspirin also reversed the expression of four important metastasis‐related genes (E2F1, VEGFA, MMP3, and CCNE1) (Table 1).

**Table 1:** Regulation of aspirin and differential genes involved in identified pathways.

**Gene**	**mRNA expression in colon cancer**	**Aspirin–mRNA interaction**
BCL2	↓	↓↑
BIRC5	↑	↓↑
BMP6	↓	↑
CCL19	↓	↑
CCL3	↑	↓
**CCNE1**	↑	↓
CD36	↓	↓↑
CD40LG	↓	↓
CDC25A	↑	↓
CXCL1	↑	↑
CXCL5	↑	↑
CXCL8	↑	↑
**E2F1**	↑	↓
FOSL1	↑	↓↑
GDF15	↑	↑
HSPA6	↑	↑
IL10	↓	↑
IL1A	↑	↑
IL1B	↑	↓↑
IL1RN	↑	↓↑
IL2	↓	↓
KLRB1	↓	↑
LIF	↑	↑
MAPK12	↑	↓
MET	↑	↑
MMP13	↑	↑
**MMP3**	↑	↓
MPO	↑	↑
NCF1	↓	↓
NGFR	↓	↑
NOX4	↑	↑
PF4	↑	↓
PPBP	↑	↓
PRKCB	↓	↓
PTGS1	↓	↓
SELP	↓	↓↑
**VEGFA**	↑	↓

### 3.7. Effect of Aspirin on Metastasis‐Related Target Genes in Colon Cancer Cells

Studies have shown that salicylate, the active metabolite of aspirin, inhibits the viability and metastasis of CRC cells [14, 15]. Therefore, in this study, we used salicylate to verify the effect of aspirin on metastasis‐related genes in colon cancer cells. The results of wound healing assay and Transwell assay showed that salicylate significantly inhibited the migration ability of colon cancer cell line LoVo cells, and these results were consistent with the results of other researchers (Figure 16a, 16b, and 16c). Then, we used RT‐qPCR assay to detect the effect of salicylate on the expression of metastasis‐related genes (E2F1, CCNE1, VEGFA, and MMP3) in LoVo cells. The results showed that salicylate could significantly inhibit the expression of metastasis‐related genes in LoVo cells (Figure 16d). Cell experiments suggested that aspirin may inhibit colon cancer metastasis by inhibiting the expression of these key genes.

Figure 16Salicylate inhibits LoVo cells migration and the expression of core target genes. (a) The chemical structure of salicylate. (b) Wound healing assay of LoVo cells, showing representative images and the corresponding quantification of migration width 24 h after salicylate treatment. (c) Transwell assay of LoVo cells, showing representative images and the quantified number of cells that migrated through the Matrigel after crystal violet staining (48‐h treatment). (d) Effect of salicylate on metastasis‐related target genes in LoVo cells, determined by RT‐qPCR assay.

**Figure:**
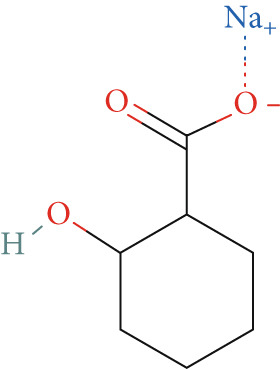
(a)

**Figure:**
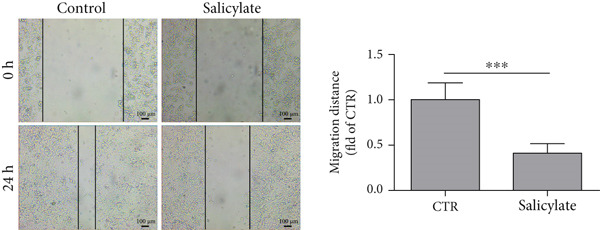
(b)

**Figure:**
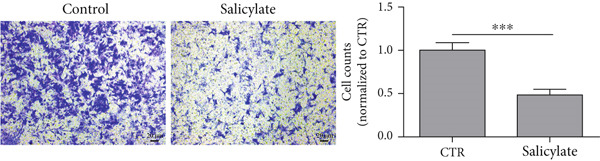
(c)

**Figure:**
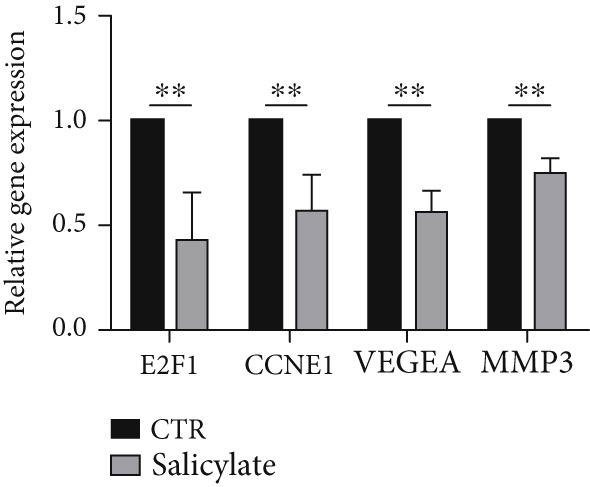
(d)

## 4. Discussion

Tumor metastasis is one of the main reasons affecting the prognosis of colorectal cancer, and its process is complex, involving multiple complex genetic and epigenetic changes [16]. Therefore, it is urgent to explore the key metastasis genes and potential regulatory mechanisms that affect the prognosis of colorectal cancer, and provide new prognostic markers or therapeutic targets for colorectal cancer.

In this study, we constructed a WGCNA co‐expression network based on 2062 metastasis‐related genes to obtain the modules most associated with colon cancer. The results showed that the blue and yellow modules were significantly associated with colon cancer, so we further analyzed the genes in these two modules. By analyzing the genes in the blue module, we found that cell cycle plays a crucial role in the tumorigenesis, recurrence, and metastasis of colon cancer. The E2F transcription factor family is one of the key epigenetic regulators in this process. Cancer is characterized primarily by infinite cell proliferation [17]. Cancer cell spread is a key aspect of metastasis and may require a switch from a proliferative to an invasive state. Recent studies have shown a functional link between cell cycle arrest and invasive activity. There is a potential link between cell cycle arrest and invasive behavior [18]. In addition, EMT is usually associated with cell metastasis and is also regulated by cell cycle. Cell behavior associated with EMT in cancer progression suggests a close association between loss of proliferation through downregulation of mitotic cell cyclin/CDK activity and upregulation of CKI [19, 20]. The yellow module’s enrichment in angiogenesis/extracellular matrix pathways aligns with NF*κ*B1/STAT3 roles in metastasis. NF‐*κ*B family transcription factors and their regulatory target genes are associated with malignant transformation, proliferation, survival, invasion/metastasis, angiogenesis, and therapeutic resistance of tumor cells [21]. The NF‐*κ*B signaling pathway promotes invasion and metastasis of NSCLC by enhancing the activation of FAK, a key kinase of focal adhesion [22]. And constitutionally activated STAT3 signaling pathways in many cancers contribute to many markers of carcinogenesis and metastasis, including promoting tumor cell proliferation and enhancing the ability of cells to migrate and invade into extracellular matrix [23]. Invasion into the extracellular matrix is one of the key steps in tumor metastasis. Multiple evidences suggest that STAT3 plays a critical role in this multi‐step process by regulating matrix metalloproteinases (MMPs). For example, activation of STAT3 in melanoma could up‐regulate MMP‐2 expression by directly binding to its promoter [24]. In addition, knockdown of STAT3 expression in pancreatic cancer cells significantly inhibited cell invasion and MMP‐7 expression [25].

Next, we established a prognostic model of colon cancer risk score by univariate and Lasso‐Cox regression analysis. The risk score model is based on 14 genes in the blue module. Among the 14 model genes, the expression levels of FZD3, CDC25C, CDCA2, NEBL, and HMMR were positively correlated with better prognosis of colon cancer. The expression levels of MTAP, GAS2, DDX11, QSOX2, AGAP6, SOX12, ATP6V1C2, STC2, and PLXNA3 were associated with poor prognosis. Most of these genes have been reported in tumors. In pancreatic cancer, overall survival (OS) of patients with high FZD3 expression was better than that of patients with low FZD3 expression [26]. In colorectal cancer, survival analysis showed that patients with high expression of NEBL had a long overall survival [27]. MTAP promotes colorectal cancer growth and metastasis through EMT [28]. UCHL1 promotes lymphatic metastasis, distant metastasis, and worse prognosis in glioma patients by enhancing GAS2 expression [29]. QSOX2 is expressed periodically in the cell cycle and transcribed by E2F1, which is essential for proliferation of NSCLC cells [30]. SOX12 is a key cancer‐related protein in many human cancers. It is associated with the progression and poor prognosis of human breast cancer, liver cancer, stomach cancer, and other tumors [31, 32]. In addition, SOX12 can promote gastric cancer metastasis by up‐regulating MMP7 and IGF1 [33]. Stalcitin 2 (STC2) is a secreted glycoprotein that regulates the progression of malignant tumors. High expression of STC2 was significantly associated with poor prognosis, lymph node metastasis, distant metastasis, and advanced clinical stage in colorectal cancer patients [34]. We constructed a nomogram model using the risk score established by these 14 genes and clinicopathological parameters to predict OS of colon cancer patients. Survival analysis and ROC curve showed that the nomogram model had good predictive performance.

Aspirin is a well‐known chemical protective agent for colorectal cancer [35, 36]. Regular aspirin use was also associated with a reduction in colorectal cancer recurrence, metastasis, and cancer‐related mortality [37]. However, the risk–benefit relationship of aspirin has not been fully explained. Finally, we explored which colon cancer patients might benefit from aspirin therapy and which patients should stop taking aspirin promptly. By analyzing 126 differentially expressed aspirin interaction genes in colon cancer, we further explored the molecular pathways that may lead to benefit or adverse outcomes in colon cancer patients treated with aspirin. The results showed that platelet activation, chemical carcinogenesis, neutrophil extracellular trap formation, cytokine–cytokine receptor interaction, IL‐17 signaling pathway, and chemokine signaling pathway were enriched. These signaling pathways contribute to tumor progression and invasiveness. Combining the expression trends of these genes in colon cancer and after aspirin treatment, we found that aspirin further upregulated NOX4, CXCL8, CXCL5, LIF, GDF15, and MMP13, while stimulated inhibition of IL2, NCF1, and PTGS1. There is evidence that the upregulation of NOX4, CXCL8, CXCL5, GDF15, and MMP13 genes in colorectal cancer promotes the metastasis of cancer cells, so a sustained upregulation of these genes after aspirin treatment will not benefit from treatment [38–41].

Importantly, aspirin also inhibited the expression of four highly expressed metastatic genes in colon cancer, including E2F1, CCNE1, VEGFA, and MMP3, which may explain its anti‐tumor and anti‐metastasis effect. Several studies have indicated that E2F1, as a transcription factor, can promote the proliferation, migration, invasion, and metastasis of colorectal cancer by regulating its target genes [42, 43]. VEGFA is known to be an angiogenic factor and is closely associated with tumor angiogenesis. Treatment targeting angiogenesis and VEGF pathways is an important component of treatment for patients with metastatic colorectal cancer [44]. MMP3 is a member of the matrix metalloproteinases family, which promotes the migration, invasion and metastasis of cancer cells by altering tumor environment, intracellular signaling pathways, and transcription [45]. As shown in Table 1, aspirin has been reported to inhibit the expression of these genes. In addition, we confirmed at the cellular level that aspirin has the ability to inhibit the metastasis of colon cancer cells and demonstrated that aspirin can suppress the expression of metastasis‐related genes (E2F1, VEGFA, MMP3, and CCNE1) in colon cancer cells. Thus, colon cancer patients with these four genes significantly down‐regulated after aspirin treatment could benefit from chemoprophylaxis.

Therefore, aspirin’s chemoprophylactic efficacy appears subtype‐dependent: Patients with aspirin‐induced downregulation of E2F1, CCNE1, VEGFA, and MMP3 may benefit, whereas persistent upregulation of NOX4, CXCL8, CXCL5, GDF15, or MMP13 may indicate non‐response. This aligns with aspirin’s known anti‐metastatic mechanisms via E2F1 [42, 43], VEGFA [44], and MMP3 [45] suppression.

## 5. Limitations

While E2F1, NF*κ*B1, and STAT3 are proposed as regulatory transcription factors, these predictions are not experimentally validated. This study analyzed colon cancer patients who might benefit from aspirin chemoprophylaxis; however, we evaluated it primarily by mapping a statistical association between gene expression regulation and aspirin. Factors such as drug dose, route of administration, duration, individual drug metabolic rate, and various colon cancer types cannot be taken into account in this process, so these need to be explored in our further in vitro and in vivo studies. This study only used the TCGA dataset for analysis and lacked external validation using independent cohorts. In future work, we will conduct further verification using platform data such as GEO, CPTAC, or clinical CRC samples.

## 6. Conclusion

In conclusion, the metastasis gene‐derived risk score is an independent prognostic factor in colon cancer. The integrated nomogram facilitates personalized survival prediction. In addition, aspirin chemoprophylaxis should be considered for patients exhibiting downregulation of E2F1, CCNE1, VEGFA, and MMP3, but may be ineffective in those with sustained upregulation of NOX4, CXCL8, CXCL5, GDF15, and MMP13.

## Disclosure

All authors contributed to the article and approved the submitted version. A preprint has previously been published ref. no. [46].

## Conflicts of Interest

The authors declare no conflicts of interests.

## Author Contributions

Jiawei Yu, Hui Zhang, and Jing Li: conception and design, financial support, and final approval of the manuscript. Jing Li and Xinyue Yu: manuscript writing, assembly of data, and data analysis and interpretation. Jingjing Shao and Xinyue Yu: data analysis and interpretation. Jing Li and Xinyue Yu contributed equally as first authors. Jing Li and Xinyue Yu contributed equally.

## Funding

This study was supported by the Nantong Municipal Health Commission Project (MS2022053, MSZ2024015, and QN2024026) and the Nantong University Clinical Special Project (2022LZ012).

## Data Availability

The data that support the findings of this study are available from the corresponding author upon reasonable request.
